# A Systematic Review on Antimicrobial Pharmacokinetic Differences between Asian and Non-Asian Adult Populations

**DOI:** 10.3390/antibiotics12050803

**Published:** 2023-04-23

**Authors:** Eko Setiawan, Menino Osbert Cotta, Jason A. Roberts, Mohd Hafiz Abdul-Aziz

**Affiliations:** 1 University of Queensland Centre for Clinical Research [UQCCR], Faculty of Medicine, The University of Queensland, Brisbane 4006, Australia; ekosetiawan.apt@gmail.com (E.S.);; 2 Department of Clinical and Community Pharmacy, Center for Medicines Information and Pharmaceutical Care [CMIPC], Faculty of Pharmacy, University of Surabaya, Surabaya 60293, Indonesia; 3 Departments of Pharmacy and Intensive Care Medicine, Royal Brisbane and Women’s Hospital, Brisbane 4029, Australia; 4 Division of Anaesthesiology Critical Care Emergency and Pain Medicine, Nîmes University Hospital, University of Montpellier, 30029 Nîmes, France

**Keywords:** Asian, antimicrobials, inter-ethnic differences, pharmacokinetics

## Abstract

While the relevance of inter-ethnic differences to the pharmacokinetic variabilities of antimicrobials has been reported in studies recruiting healthy subjects, differences in antimicrobial pharmacokinetics between Asian and non-Asian patients with severe pathologic conditions require further investigation. For the purpose of describing the potential differences in antimicrobial pharmacokinetics between Asian and non-Asian populations, a systematic review was performed using six journal databases and six theses/dissertation databases (PROSPERO record CRD42018090054). The pharmacokinetic data of healthy volunteers and non-critically ill and critically ill patients were reviewed. Thirty studies on meropenem, imipenem, doripenem, linezolid, and vancomycin were included in the final descriptive summaries. In studies recruiting hospitalised patients, inconsistent differences in the volume of distribution (V_d_) and drug clearance (CL) of the studied antimicrobials between Asian and non-Asian patients were observed. Additionally, factors other than ethnicity, such as demographic (e.g., age) or clinical (e.g., sepsis) factors, were suggested to better characterise these pharmacokinetic differences. Inconsistent differences in pharmacokinetic parameters between Asian and non-Asian subjects/patients may suggest that ethnicity is not an important predictor to characterise interindividual pharmacokinetic differences between meropenem, imipenem, doripenem, linezolid, and vancomycin. Therefore, the dosing regimens of these antimicrobials should be adjusted according to patients’ demographic or clinical characteristics that can better describe pharmacokinetic differences.

## 1. Introduction

The recent surge in multi-drug-resistant pathogens, combined with the shortage of new antimicrobials, has created the need to optimise the use of current antimicrobials, particularly in countries with limited resources [1,2,3,4]. Applying pharmacokinetic/pharmacodynamic (PK/PD) principles to guide antimicrobial dosing can increase the likelihood of achieving optimal PK/PD exposures, which have been associated with therapeutic success and may limit the emergence of antimicrobial resistance [5,6,7,8]. However, difficulties arise when attempting to optimise antimicrobial dosing in special patient populations that are critically ill.

Key antimicrobial pharmacokinetic (PK) parameters of critically ill patients, particularly those with sepsis, may differ from those of non-critically ill patients [9,10,11,12,13]. Critically ill patients commonly demonstrate extreme physiological changes that can alter antimicrobial PK and exposures [9,10,13]. The volume of distribution (V_d_) and drug clearance (CL) are important PK parameters that determine dosing requirements, and both may be dramatically altered during critical illness [9,12,13]. Despite profound physiological and PK differences, critically ill patients in the ICU typically receive conventional antimicrobial dosing, which may likely lead to suboptimal antimicrobial exposure and therapeutic failure in this patient population. Therefore, antimicrobial dosing adjustment is needed to ensure that the optimal PK/PD exposures are achieved.

In addition to the acute illness-mediated changes in PK, inter-ethnic differences may also contribute to differences in PK parameters. The influence of ethnicity on drug PK has been reported for cyclosporine [14], methadone [15], efavirenz [16], tacrolimus [17], warfarin [18], nifedipine [19], midazolam [20], and mycophenolic acid [21]. Inter-ethnic PK differences may stem from differences in (1) body composition [22,23,24] or protein binding capacity [25,26]; (2) metabolic capacity due to genetic polymorphism in CYP450 [27,28]; and (3) the drug elimination process due to the variability in genes responsible for drug transporters, particularly those for biliary excretion [29,30,31].

The clinical relevance of inter-ethnic differences to antimicrobial PK variability was reviewed by Tsai et al. in 2015 [32]. However, the relevance of inter-ethnic PK differences in patients with severe pathologic conditions (e.g., critically ill patients with sepsis) has not been reviewed thus far; Tsai et al. only included studies that recruited healthy volunteers in their systematic review. In addition, the systematic review mostly included studies of orally administered antimicrobials, whereas critically ill patients in the ICU almost always receive intravenous antimicrobials [32].

Asia is the region with the largest population and diverse ethnic groups [33]. Describing the PK of antimicrobials in the Asian population is important to determine dosing requirements for this population. Numerous countries in this region rely heavily on product information to guide antimicrobial dosing, which can potentially be flawed for critically ill patients [34,35,36]. Furthermore, dosing recommendations from the product information were mainly derived from dose-finding studies that mainly included subjects from non-Asian populations [37,38]. These doses may not always provide the same exposure for Asian patients due to potential inter-ethnic PK differences [32].

The aim of this systematic review is to describe potential differences in antimicrobial PK between Asian and non-Asian populations with reference to data from healthy volunteers and non-critically ill and critically ill patients.

## 2. Results

### 2.1. Study Selection

**Figure 1:**
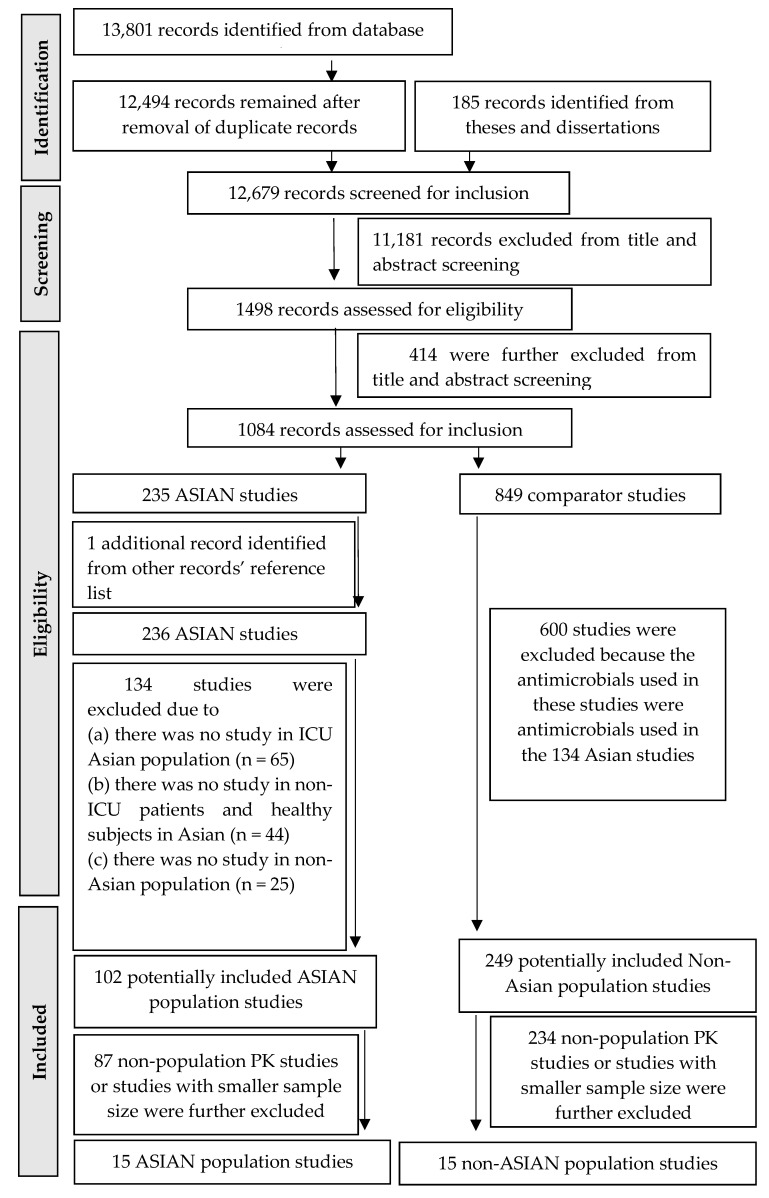
Diagram for selecting studies.

The initial literature search from the journal and theses/dissertation databases identified 12,494 and 185 records, respectively. After removing duplicate records (*n* = 1307) and irrelevant records (*n* = 11,595), 1084 records were assessed in accordance with the inclusion and exclusion criteria. Only 102 and 249 records in Asian and non-Asian populations, respectively, were further selected for a full review. Of these, 15 Asian and 15 non-Asian PK studies were included in the final review [39,40,41,42,43,44,45,46,47,48,49,50,51,52,53,54,55,56,57,58,59,60,61,62,63,64,65,66,67,68]. The full process of study selection in this systematic review is described in Figure 1. The Asian PK studies were from seven countries, including Japan (*n* = 5), Thailand (*n* = 3), South Korea (*n* = 2), India (*n* = 2), China (*n* = 2), and Malaysia (*n* = 1). The population PK analysis was performed in 22 out of 30 studies.

The quality of studies included in this systematic review was assessed in accordance with the ClinPK checklist (Supplementary Table S1). All items required in the title/abstract and background sections are well reported by at least 90% of studies except for one requirement, which is to “report the PK parameters of the studied antimicrobials known in the literature” (63%). In the methods section, limited studies (33.3%) provided information on potential drug interactions with the studied antimicrobials. Only 11 (36.7%) studies clearly described the specific body weight descriptor used to determine the dose of antimicrobials or to calculate the PK parameters. In the results section, three requirements are reported by at least 90% of studies: (1) reporting the variables that may influence PK variabilities (93.3%); (2) including the measures of precision for the reported PK parameters (90%); and (3) the applicability of study findings (100%).

Studies included in the final PK comparison were on meropenem [39,40,41,42,43,44], imipenem [45,46,47,48,49,50], doripenem [51,52,53,54,55,56], linezolid [57,58,59,60,61,62], and vancomycin [63,64,65,66,67,68] (Table 1). Population PK studies in this systematic review identified body weight as a significant determinant of V_d_ for hydrophilic antimicrobials, including meropenem [42,43], imipenem [50], doripenem [51,55,56], linezolid [59,62], and vancomycin [63,66,68]. Renal function was found to be a significant predictor of CL for all studied antimicrobials included in the systematic review [41,42,43,44,47,50,53,55,56,59,63,65,66,67,68]. *Serum creatinine (Se_Cr_)* was found as a significant covariate of CL in one study for meropenem [41]. Other covariates that have been reported to also influence the V_d_ and CL of these antimicrobials are listed in Table 2.

### 2.2. Pharmacokinetic Differences between Asian and Non-Asian Population Groups

Table 3 summarises the antimicrobial PK parameters between Asian and non-Asian populations. The influence of ethnicity on primary PK parameters (i.e., V_d_ and CL) for each antimicrobial is further discussed below. Table 4 summarises the observed inter-ethnicity differences in V_d_ and CL for each antimicrobial.

#### 2.2.1. Carbapenems

##### Meropenem

Inter-ethnic differences in V_d_ for meropenem between Asian and non-Asian patients were not observed. Studies in healthy subjects reported a relatively comparable meropenem V_d_ between Asian and non-Asian subjects. The reported mean V_d_ of meropenem in Asians and non-Asians was 0.16–0.20 L/kg and 0.18–0.19 L/kg (Table 3), respectively, depending on the dose administered [39,40]. Additionally, inconsistent findings between ICU and non-ICU studies may suggest that factors other than ethnicity could be driving these V_d_ differences [41,42,43,44]. In non-ICU patients, the mean V_d_ of meropenem in the central compartment was 4 times larger in Asian patients when compared with non-Asian patients (0.64 L/kg versus 0.15 L/kg; Table 3) [41,42]. However, in ICU patients, the mean V_d_ of meropenem in the central compartment was 2 times larger in non-Asian than in Asian patients (0.34 L/kg versus 0.16 L/kg; Table 3) [43,44].

Likewise, inter-ethnic differences in CL for meropenem between Asian and non-Asian patients were also not observed. Studies recruiting healthy subjects [39,40], non-ICU patients [41,42], and ICU patients (450, 451) reported comparable mean CL of meropenem between Asians and non-Asians (Table 3). The difference in the mean CL of meropenem between Asians and non-Asians among healthy subjects was up to 0.04 L/h/kg, which is similar to those of ICU studies [39,40,43,44]. In non-ICU studies, however, the difference was 0.01 L/h/kg.

##### Imipenem

Inter-ethnic differences in V_d_ for imipenem appear to be unlikely, as only ICU studies showed distinct V_d_ differences between Asian and non-Asian patients. In ICU studies, the mean V_d_ of imipenem in Asian and non-Asian patients was 0.5 L/kg and 0.38 L/kg, respectively (Table 3 and Table 4) [49,50]. In healthy subjects, the difference in the means of imipenem V_d_ between Asians and non-Asians was around 0.03–0.06 L/kg, depending on the dose administered, whilst it was 0.02–0.03 L/kg in non-ICU studies (Table 3) [45,46,47,48].

The available data do not suggest inter-ethnic differences in imipenem CL between Asian and non-Asian populations. Even though ICU studies highlighted that the mean CL of imipenem among Asian patients was double that among non-Asian patients (0.39 L/h/kg versus 0.16 L/h/kg; Table 3) [49,50], this observation was not seen in studies conducted in healthy subjects [45,46] and non-ICU patients [47,48]. The mean CL of imipenem was relatively comparable between Asians and non-Asians in studies of healthy subjects and non-ICU patients.

##### Doripenem

The difference in V_d_ for doripenem between healthy Asian and non-Asian subjects may suggest an inter-ethnic influence [51,52]. The mean total V_d_ of doripenem was larger in Asian healthy subjects when compared with non-Asian healthy subjects (0.31 L/kg versus 0.21 L/kg; Table 3). In addition to this observation, a remarkable difference was found in the mean V_d_ in the central compartment between Asian and non-Asian healthy subjects (0.26 L/kg versus 0.13 L/kg; Table 3). However, both non-ICU and ICU studies reported that the mean V_d_ of doripenem was comparable between Asian and non-Asian patients. The differences in the mean V_d_ of doripenem between Asians and non-Asians were 0.09 L/kg and 0.07 L/kg in non-ICU and ICU studies, respectively (Table 3) [53,54,55,56].

In contrast to V_d_, inter-ethnic CL differences for doripenem might not be supported. Even though the mean CL of doripenem in Asian healthy subjects was 0.15 L/h/kg higher than that of non-Asian subjects (Table 3), caution should be exercised to support inter-ethnic differences because the study in non-Asian healthy subjects did not report detailed patient characteristics, including age and renal function [52]. It is also worth mentioning that both studies recruited healthy volunteers with different age ranges, which may have influenced this observation [51,52]. The non-ICU and ICU studies consistently suggested that non-Asian patients had faster doripenem CL when compared with Asian patients [53,54,55,56]. This could be related to the fact that the mean value of renal function in non-Asian patients, in both non-ICU and ICU settings, was higher than that in Asian patients.

#### 2.2.2. Oxazolidinones

Linezolid is the only oxazolidinone studied in this systematic review. The oral bioavailability of linezolid was reported in one study, which reported a value of approximately 93% [57]. Some studies included in our systematic review demonstrated that linezolid was eliminated through both non-renal and renal routes [58]. However, almost all of the included studies in this systematic review reported linezolid CL as the total CL rather than as renal and non-renal CL separately [57,59,60,61,62].

##### Linezolid

Inter-ethnic differences were not found for the V_d_ of linezolid. When Asians and non-Asians were compared, healthy subjects [57,58] and non-ICU patients [59,60] showed a similar mean value of V_d_. In healthy subjects, the mean V_d_ of linezolid in Asians and non-Asians was reported as 0.65–0.67 L/kg and 0.58–0.61 L/kg (Table 3), respectively, depending on the route of administration [57,58]. From non-ICU studies, the reported mean V_d_ of linezolid in Asian and non-Asian patients was 0.59 L/kg and 0.58 L/kg, respectively [59,60]. However, ICU studies showed a remarkable difference in the mean V_d_ of linezolid in the central compartment between Asian and non-Asian patients (0.34 L/kg versus 0.19 L/kg; Table 3) [61,62]. This difference may be due to factors other than inter-ethnic differences.

Similar to V_d_, inter-ethnic differences in linezolid CL were not observed. All studies involving healthy subjects and non-ICU and ICU patients reported a relatively similar mean CL of linezolid between Asian and non-Asian populations (Table 3) [57,58,59,60,61,62].

#### 2.2.3. Glycopeptide

##### Vancomycin

The studies included in this systematic review did not demonstrate a pattern of inter-ethnic V_d_ differences between Asian and non-Asian populations for vancomycin. Studies of healthy subjects [63,64] showed a relatively comparable V_d_ between Asians and non-Asians. It is worth mentioning that comparisons of V_d_ in studies of healthy subjects could only be performed for the V_d_ in the central compartment because the peripheral compartment value was not reported in non-Asian studies [64]. The difference in the mean V_d_ of vancomycin in the central compartment between Asian and non-Asian healthy subjects was between 0.07 and 0.1 L/kg, depending on the dose of vancomycin [63,64]. In non-ICU studies, the mean V_d_ of vancomycin in the central compartment in Asian patients was more than 2 times larger than that in non-Asian patients (0.73 L/kg and 0.28 L/kg; Table 3) [65,66], while in ICU studies, the mean V_d_ of vancomycin in the central compartment was 4 times larger in non-Asian than in Asian patients (1.53 L/kg versus 0.40 L/kg; Table 3). Inconsistent findings between non-ICU and ICU studies may indicate the influence of factors other than ethnicity [67,68].

Inter-ethnic differences were also not observed for vancomycin CL. Based on studies of healthy subjects, non-ICU patients, and ICU patients, the mean CL of vancomycin was relatively comparable between Asian and non-Asian populations [63,64,65,66,67,68]. The mean CL differences between Asians and non-Asians were 0.02 L/h/kg, 0.03–0.02 L/h/kg (depending on the site of infection), and 0.01 L/h/kg in healthy subjects, non-ICU patients, and ICU patients, respectively (Table 3) [63,64,65,66,67,68].

## 3. Discussion

In this systematic review, we reviewed inter-ethnic PK differences for select antimicrobials between adult Asian and non-Asian populations, including healthy subjects and ICU, and non-ICU patients [39,40,41,42,43,44,45,46,47,48,49,50,51,52,53,54,55,56,57,58,59,60,61,62,63,64,65,66,67,68]. We found limited evidence to support the hypothesis that Asian subjects differ from non-Asian subjects in the PK disposition of carbapenems, vancomycin, and linezolid.

We found studies recruiting healthy subjects, non-ICU patients, and ICU patients on meropenem, imipenem, and doripenem in Asian and non-Asian populations. Of the three carbapenems, inter-ethnic differences could only be suggested for the V_d_ of doripenem. The V_d_ differences between meropenem and imipenem were only observed in studies involving hospitalised patients (ICU and non-ICU), and the findings were inconclusive to show which ethnic groups had a larger V_d_. Therefore, factors other than ethnicity, such as demographic (e.g., age) or clinical (e.g., sepsis) factors, are suggested to contribute to the V_d_ differences between meropenem and imipenem [41,42,43,44].

In non-ICU studies, the mean V_d_ of meropenem in the central compartment was 4 times larger in Asian patients when compared with non-Asian patients (0.64 L/kg versus 0.15 L/kg) [41,42]. However, it could be possible that the larger V_d_ in non-ICU Asian patients was more likely to be related to age differences rather than ethnicity. Patients included in the Asian study were older compared to the non-Asian study (71.5 ± 13.5 versus 39.6 ± 18.2 years old). Mattioli et al., in their study, highlighted that the V_d_ of meropenem tended to be larger among older patients, specifically after 61 years old, which supports our findings [69]. Similar to the non-ICU studies, some caution should be exercised when interpreting the V_d_ differences observed in ICU studies. In studies involving ICU patients, the mean V_d_ of meropenem was 2 times larger in non-Asian than in Asian patients (0.34 L/kg versus 0.16 L/kg) [43,44]. The mean age of ICU patients in the non-Asian study [44] was 63.26 years old, while the Asian study (450) did not report the age of participants. If the Asian study predominantly recruited patients with a younger age, then a lower V_d_ should be expected.

Similar to meropenem, V_d_ differences for imipenem may not indicate an inter-ethnic influence since the threshold supporting a “distinct difference” (≥0.1 L/kg) was only observed in ICU studies [49,50]. The mean V_d_ of imipenem in Asian ICU patients was larger than that in non-Asian ICU patients, and this could be attributed to several possible factors. First, serum albumin concentrations could contribute to the imipenem V_d_ differences, as serum albumin was found as a significant covariate influencing the V_d_ of imipenem [50,70,71,72]. Unfortunately, the serum albumin data were not reported in the Asian ICU study, and further analysis to rule out the influence of serum albumin could not be performed [49]. Second, the V_d_ of hydrophilic antimicrobials in critically ill patients, including imipenem, is subjected to extravascular fluid changes [9]. The third spacing phenomenon due to capillary leakage syndrome, which is commonly reported in critically ill patients with sepsis, might also contribute to the increase in V_d_ for imipenem [9]. In addition, the aggressive fluid treatment to overcome hypotension during septic shock in critically ill patients could further contribute to V_d_ increases [9,73,74,75]. Eighteen out of fifty-one patients in the non-Asian ICU study were patients with septic shock. However, no information was available regarding disease severity (including septic shock) in the Asian ICU study. If the number of patients with septic shock was higher in the Asian ICU study, then this could have contributed to the larger V_d_.

Possible inter-ethnic V_d_ differences can only be suggested for doripenem, as a larger V_d_ in healthy Asian subjects was observed when compared with non-Asian subjects [51,52]. One plausible factor contributing to this finding is that the extracellular water volume per kg body weight is larger in Asian subjects [24,26]. Findings in the other two carbapenems also showed that V_d_ per kg of weight was higher in Asian than non-in Asian healthy subjects, even though the difference might not reach the threshold defined in our study [39,40,45,46]. The mean V_d_ of meropenem and imipenem in Asian healthy subjects was 0.01 L/kg and 0.02–0.04 L/kg higher when compared to non-Asian healthy subjects, respectively [39,40,45,46].

We did not observe inter-ethnic differences in CL for doripenem, even though the difference in the means of CL between Asian [51] and non-Asian healthy subjects (0.15 L/h/kg) [52] surpassed the threshold to indicate a “distinct difference” (≥0.1 L/h/kg) [32]. The main reason for this is due to the absence of data regarding the central tendency (such as mean and median values) for renal function in the non-Asian study [52]. Comparable renal function could not be assumed because the non-Asian study included older healthy subjects (up to 65 years old), while the Asian study recruited healthy subjects aged ≤ 30 years old. It is worth mentioning that, in addition to ethnicity, the renal blood flow and glomerular filtration rate are also influenced by age [76,77,78,79,80]. Compared to subjects aged below 40 years old, significant reductions in renal blood flow and glomerular filtration were found in subjects aged above 55 years old [79,80]. Therefore, lower doripenem CL in the non-Asian study could also be related to the recruitment of subjects with a wide range of ages.

For non-carbapenem antimicrobials, including linezolid and vancomycin, studies recruiting hospitalised patients showed remarkable V_d_ differences [61,62,67,68]. The absence of inter-ethnic PK differences for linezolid and vancomycin among healthy subjects was also emphasised in a previous systematic review by Tsai et al. [32]. In ICU studies, considerable V_d_ differences for linezolid were observed in the central compartment rather than in the peripheral compartment [61,62]. The reported mean V_d_ in the central compartment in Asian and non-Asian ICU studies was 0.34 L/kg and 0.19 L/kg, respectively, while the reported mean V_d_ in the peripheral compartment was 0.39 L/kg and 0.34 L/kg, respectively [61,62]. It should be emphasised that the Asian ICU study was conducted specifically among patients with sepsis and septic shock, while the majority of patients in the non-Asian study were pneumonia patients. The number of patients with sepsis in the non-Asian study, however, was unknown. As a hydrophilic antimicrobial [81], even though some authors also classified linezolid as an antimicrobial with moderately lipophilic properties [82,83], the V_d_ of linezolid in the central compartment may potentially increase due to aggressive fluid therapy to overcome the consequences of sepsis and septic shock [74,75]. Thus, the larger V_d_ for linezolid in the Asian study when compared to the non-Asian study could be more related to the severity of illness rather than inter-ethnic differences.

Based on the ICU studies, the mean V_d_ of vancomycin in Asian patients was approximately half that in non-Asian patients (0.40 L/kg versus 1.53 L/kg) [67,68]. Similar to what we suggested for linezolid, the different mean V_d_ of vancomycin reported in ICU studies could be more related to the severity of illness rather than the influence of ethnicity. One population PK study in our systematic review emphasised that the V_d_ of vancomycin in patients with sepsis and pneumonia was larger than in those with other types of infections [63]. It is worth noting that approximately 20% of patients in the Asian study were diagnosed with conditions other than pneumonia or sepsis, while patients in the non-Asian study exclusively recruited patients with sepsis. The severity of illness might also contribute to the difference in the mean V_d_ of vancomycin in non-ICU studies. However, no non-ICU studies in either Asian or non-Asian patients reported detailed patient and clinical characteristics, including the patients’ diagnoses [65,66]. In addition to the severity of illness, different times of collecting blood samples might also contribute to the larger V_d_ in Asians in the non-ICU study [84]. The blood samples in the non-ICU study [65] were collected after the fifth dose of vancomycin, indicating a steady-state condition. In the comparator study, the time of sample collection was not reported [66].

There are several limitations in this systematic review. Firstly, we included only one study to represent and compare the Asian and non-Asian populations. Therefore, we might not be able to adequately explain the PK variability for each antimicrobial in all Asian and non-Asian populations. Secondly, detailed information about ethnicity might not always be available in non-Asian studies, and it could be possible that the non-Asian population in the reviewed studies included Caucasian, Hispanic, and African-American subjects. Moreover, in the era of rapid globalisation, it is also possible that the non-Asian studies in our systematic review included some percentage of Asian subjects. However, such detailed information about ethnicity might not always be possible to gather from each study. Therefore, the findings might be different if comparisons were made specifically between two distinct ethnicities, for example, between Indonesians and African-American subjects. Thirdly, it is important to note that we included studies with different PK compartment models, and this could limit the interpretation of PK differences, particularly V_d_. Many factors, including the number of samples, the time of blood sampling, and the PK analysis method, could contribute to the different reported PK models; it is indeed challenging to find studies with such comparable factors. In one compartment, the distribution of antibiotics was assumed to be rapid and homogeneous among all organs, which might not always be true for all antibiotics, especially for lipophilic antibiotics. On the other hand, in the two-compartment model, the distribution of antibiotics intravascularly and among organs with equilibrium was represented as V_c_, and the distribution among organs with late equilibrium was represented as V_p_. Although the interpretation of inter-ethnic V_d_ differences in the present systematic review should be made with caution, comparing the V_d_ of the one-compartment model with the V_c_ of the two-compartment model could be considered a reasonable approach for hydrophilic antibiotics, as these antibiotics were not distributed to the intra-cellular compartment. Furthermore, effort to make a rigorous PK comparison was made by considering not only the PK compartment model but also other factors influencing the PK parameters. Finally, it should be noted that limited inter-ethnic PK differences in our systematic review were observed for antimicrobials that undergo minimal hepatic metabolism via the CYP450 system. As human physiology is indeed influenced by ethnicity, particularly CYP-mediated drug metabolism, PK differences should be anticipated among patients with different ethnicities [27,76,77,78].

## 4. Materials and Methods

This systematic review was conducted according to the Preferred Reporting Items for Systematic Reviews and Meta-analyses (PRISMA) [85]. The 27-item PRISMA checklist of this systematic review can be found in Supplementary Materials (Tables S2 and S3).

### 4.1. Search Strategy

Six journal databases (Pubmed, Embase, Web of Science, Scopus, CINAHL, and Joanna Briggs Institute (JBI) EBP Database) and six theses/dissertation databases (Networked Digital Library of Theses and Dissertations, Open Access Theses and Dissertations, Hong Kong University Theses Online, China Doctoral Dissertations Full-Text Database, and National University of Singapore Theses, Online Union Catalogue of Indian Universities) were searched from inception to September 2017. The search was updated in February 2021. ES developed a list of search terms with additional inputs from other co-authors. There were two stages of the literature search in this study.

The first stage aimed to identify all PK studies in the Asian population. Boolean operators “AND” and “OR” of several pre-defined search terms were used in the first stage of the literature search (Supplementary Table S4). The search terms used in all databases can be found in the PROSPERO record (registration number CRD42018090054). The second stage aimed to identify PK studies in the non-Asian population, which were used as comparator studies. No restriction was applied in either stage of the literature search. The cited references of relevant articles were checked to find additional PK studies in both Asian and non-Asian populations.

### 4.2. Study Selection

Titles and abstracts from the search were combined and screened in EndNote X9 by ES. All duplicates and irrelevant studies were removed from the database, and the remaining titles and abstracts were reviewed in accordance with the inclusion criteria. Full-text articles of all potentially eligible studies were retrieved and further assessed based on the inclusion criteria. Any uncertainty regarding study inclusion was solved by discussion with co-authors (MOC and MHAA).

Studies that reported V_d_ and/or CL and recruited subjects ≥18 years old were included in this systematic review. For each antimicrobial, PK studies of healthy subjects and non-critically ill and critically ill patients from both Asian and non-Asian populations were selected. The study with the largest sample size that applied a population PK approach [86,87,88] was selected if more than one study was available. A comparison of PK parameters was not made for an antimicrobial if we could not find at least one published study for each population of interest. Studies were excluded if they met at least one of the following criteria: (1) only recruited patients on RRT and/or extracorporeal membrane oxygenation (ECMO); (2) specifically conducted in burn patients; (3) presented PK parameters of a nebulised antimicrobial; (4) available only as an abstract of a poster presentation; (5) was a case report including only one patient; and (6) published in languages other than English.

### 4.3. Data Extraction and Quality Assessment of Pharmacokinetic Studies

Data extraction was performed by ES, and the following information was extracted from each study: (1) study design (population PK or non-population PK study); (2) number of subjects, (3) number of PK compartments; (4) subject/patient characteristics (age in years, body weight in kg, and CL_Cr_ in mL/min or mL/min/1.73 m^2^); (5) antimicrobial dosing regimen; (6) PK sampling information (after single dose, multiple doses, or both); and (7) PK parameters. The following PK parameters (abbreviation; unit) were extracted: (1) CL (L/h), (2) t_1/2_ (h), (3) V_c_ (L), and V_p_ (L); (4) intercompartmental clearance (Q; h^−1^); (5) K_CP_ (h^−1^) and K_PC_ (h^−1^). In addition to these PK parameters, the interindividual variability (IIV) for each PK parameter was extracted for population PK studies. Relevant adjustments were performed if the PK parameters were presented in different units. Another author (MHAA) performed a random check on 10% of the manuscripts to ensure the accuracy of data extraction.

The quality of the included studies was reviewed according to the consensus-based checklist for reporting PK studies [89]. The ClinPK checklist consisted of 24 items, which evaluated the robustness of a PK report in six (6) domains: (1) title/abstract (2 items); (2) background (3 items); (3) methods (10 items); (4) results (6 items); (5) discussion/conclusion (2 items); and (6) other information (1 item).

### 4.4. Pharmacokinetic Comparison Analysis

Patient characteristics and PK parameters are presented as means (with or without standard deviation, SD) and ranges. Original data presented as medians were converted to means by using the equation developed by Luo et al. (2018) [90]. The Cockcroft–Gault equation was used to calculate the CL_Cr_ if a study provided the Se_Cr_ concentration [91]. Comparisons of V_d_ and CL between Asian and non-Asian populations were performed as per kg body weight. Values of 60 kg and 70 kg were used as adjustments in Asian and non-Asian populations, respectively, for a study without body weight data [92,93,94,95].

In this systematic review, a difference in V_d_ or CL for an antimicrobial was classified as “were observed”, meaning influenced by ethnicity, if these differences were (1) consistently found in studies of healthy subjects, non-ICU patients, and ICU patients or (2) observed in studies recruiting healthy subjects with relatively comparable patient demographic characteristics. We set a difference value of ≥0.1 for both V_d_ and CL as the threshold to define inter-ethnic differences, as also used in a previous systematic review [32].

## 5. Conclusions

Inconsistent differences in the PK of carbapenems, vancomycin, and linezolid between Asian and non-Asian subjects/patients in this systematic review indicate that ethnicity might not be a good proxy to adjust the dosing regimens for the studied antimicrobials. Moreover, the findings among studies of healthy subjects further suggested that the PK disposition of carbapenems, vancomycin, and linezolid in Asian subjects were relatively comparable to that in non-Asian subjects. Thus, dose adjustments for these antimicrobials should be made according to patients’ demographic or clinical characteristics that can better describe PK differences. A better understanding of significant covariates explaining the PK variabilities of antimicrobials could be helpful to identify which patients will benefit the most from the implementation of therapeutic drug monitoring (TDM). Finally, knowledge on the distribution of the minimum inhibitory concentrations (MICs) of important pathogens in a specific area would further maximise the attainment of PK/PD exposures associated with therapeutic success.

## Figures and Tables

**Table 1:** Pharmacokinetic studies of antimicrobials meeting inclusion criteria.

Author (Year)	Type of Study (POP PK Software)	Population Included in the Study (n)	Dosage Regimen; Route of Administration; Timing of Blood Sampling	Age (Years)	Weight (Kg)	Clearance Creatinine (CLCr; mL/min)	PK Parameters *
Meropenem							
Jaruratanasirikul et al. (2003) [39]	Non-POP PK (NA)	Healthy subjects (12)	500–1000 mg Bolus (10 min) and 3 h infusion Single dose	32.58 ± 8.94 (18–48)	59.69 ± 7.83 (45–72)	NI	V_d_, CL, t_1/2_
Krueger et al. (2005) [40]	POP PK (NPAG)	Healthy subjects (16)	Low dose group: 0.5 g 0.5 h infusion Q8 h or three doses of 0.5 g as CoI after LD of 0.25 g High dose group: double dose of low dose group Multiple doses	22.5 ^c^	66.6 ^c^	NI	V_c_, CL, K_CP_, K_PC_
Muro et al. (2011) [41]	POP PK (NONMEM)	Japanese Patients ^a^ (68)	NI about dose and route administration Multiple doses	71.5 ± 13.5 (25–91)	52.1 ± 13.9 (30.7–107)	65.5 ± 55.8 (8.8–406)	V_d_, CL
Li et al. (2006) [42]	POP PK (NONMEM)	Patients with IAI, CAP, VAP (79)	05–2 g 0.5 to 3 h infusion Multiple doses	39.6 ± 18.2 (18–93)	73.0 ± 16.1 (40.6–127)	93	V_d_, CL, t_1/2_
Mathew et al. (2016) [43]	POP PK(Pmetrics)	ICU patients with sepsis, polytrauma (35)	500–1000 mg bid or tid 0.5 to 3 h infusion Multiple doses	NI	NI	65.08 ± NI (10–134.8)	V_c_, K_e_, K_CP_, K_PC_
Idoate grijalba et al., (2019) [44]	POP PK(Pmetrics)	ICU Patients (80)	Multiple doses	63.26 ± 15.07	72.76 ± 19.17	^b^	V_d_, K_e_
Imipenem							
Jaruratanasirikul et al. (2005) [45]	Non-POP PK (NA)	Healthy subjects (8)	500 mg or 1000 mg 0.5 to 2 h infusion Q6 h Multiple doses	28.25 ± 4.98 (24–39)	58.75 ± 8.61 (51–75)	NI	V_d_, CL, t_1/2_
Norrby et al. (1983) [46]	Non-POP PK (NA)	Healthy subjects (16)	500–1000 mg with or without cilastatin Q8 h 20 min infusion Multiple doses	25 ± NI (18–40)	75 ± NI (60–89)	NI	V_c_, CL, t_1/2_, K_CP_, K_PC_
Yoshizawa et al. (2012) [47]	POP PK (NONMEM)	Patients with renal impairment (27)	500 mg with 500 mg cilastatin (NI) 0.5 to 1 h infusion Single dose	59.12 ^b^	58.76 ^b^	57.95 ^b^	V_c_, V_p_, CL_ren_, CL_non-ren_, Q
Finch (1986) [48]	Non-POP PK (NA)	Elderly Patients with acute LRTIs (6)	500 mg with 500 mg cilastatin Q6 h Over 0.5 h infusion Multiple doses	76.7 (68–83)	63.3 (49.8–76)	50.6 (31–80)	V_d_, CL, t_1/2_
Abhilash et al. (2015) [49]	Non-POP PK (NA)	ICU patients with renal, pulmonary, gastrointestinal, and skin infections (30)	1 g Q8 h Over 40 min infusion Multiple doses	43 ± NI (23–81)	64.1 ± 10.74 (38–83)	NI (30–181)	V_d_, t_1/2_
Couffignal et al. (2014) [50]	POP PK (Monolix)	ICU patients with pneumonia (51)	500–1000 mg Q8 h 0.5 h infusion Multiple doses	59.31 ^b^	78.47 ^b^	121.72 ^b,d^	V_c_, V_p_, CL, Q
Doripenem							
Kim et al. (2018) [51]	POP PK (NONMEM)	Healthy subjects (11)	250 mg 1 h infusion Single dose	25 Range (22–30)	60.9 Range (50–80)	122 Range (101–139)	V_c_, V_p_, CL, Q
Bhavnani et al. (2005) [52]	POP PK (NONMEM)	Healthy subjects (24)	500–1000 mg Q8–12 h IV Multiple doses	NI (18–65)	NI	NI	V_c_, V_p_, CL, Q
Lee et al. (2017) [53]	POP PK (NONMEM)	Patients with pyelonephritis, IAI, neutropenic fever, sepsis (37)	250 mg or 500 mg Q8 h 1 h infusion Multiple doses	61.7 ± 17.9 (NI)	59.8 ± 12.4 (NI)	66.7 ± 34.4 (NI)	V_d_, CL
Bhalodi et al. (2013) [54]	POP PK (big NPAG)	Non-ICU patients ^a^ (12)	250–2000 mg Q8–12 h 1 to 4 h infusion Multiple doses	59.7 ± 18.7 (NI)	96.2 ± 40.8 (NI)	98.8 ± 55.3 (15–221)	V_c_, V_d_, CL, K_CP_, K_PC_
Abdul-Aziz et al. (2016) [55]	POP PK (NONMEM)	ICU patients with IAI, sepsis, VAP (12)	500 mg Q8 h 1 h infusion Mixed single and multiple doses	47.97 ^b^	NI	83.9 ^b^	V_c_, V_p_, CL, Q
Roberts et al., (2013) [56]	POP PK (NONMEM)	ICU patient with nosocomial pneumonia (31)	250 or 500 mg 0.5 to 4 h infusion Multiple doses	57.8 ± 14.9 (NI)	83.0 ± 19.0 (NI)	137 ± 71 (NI)	V_c_, V_p_, CL, Q
Linezolid							
Yang et al. (2017) [57]	Non-POP PK (NA)	Healthy subjects (22)	600 mg Oral or 1 h infusion Single dose	28.27 ± 2.21 (NI)	66.73 ± 4.15 (NI)	NI	V_d_, CL, t_1/2_
Stalker et al. (2003) [58]	Non-POP PK (NA)	Healthy subjects (30)	375–625 mg Q12 h Oral and IV Single and multiple doses	625 mg = 33.0 (21.7–48.0) IV 625 mg = 24.8 (19.0–33.0)	625 mg = 73.3 (54.2–80.7) IV 625 mg = 79.4 (67.6–94.6)	NI	V_d_, CL, CL_ren_, Cl_non-ren_, t_1/2_
Sasaki et al. (2011) [59]	POP PK (NONMEM)	Patients with infectious diseases ^a^ (50)	300–600 mg bid 1 to 2 h infusion Multiple doses	69.1 ± 12.8 (32–92)	57.3 ± 12.1 (38.4–100)	74.0 ± 54.5 (9.43–330)	V_d_, CL
Crass et al. (2019) [60]	POP PK (NONMEM)	Adult patients with oral and/or intravenous linezolid (603)	600 mg Q12 h Oral or IV Number of doses: NI.	62 ± 15	76 ± 19	81 ± 39	V_d_, Cl_non-ren_, Cl_ren_, K_a_
Ide et al. (2018) [61]	POP PK (NONMEM)	ICU patients with sepsis or septic shock (17 divided into two groups ^#^)	600 mg Q12 h 1 h infusion Multiple doses	Group 1: 65.1 ± 14.5 Group 2: 74.3 ± 11.3	Group 1: 57.8 ± 7.54 Group 2: 53.4 ± 10.2	Group 1: 98.1 ^b^ Group 2: 20.59 ^b^	V_c_, V_p_, CL, Q
Taubert et al. (2016) [62]	POP PK (NONMEM)	ICU patients with ARDS, peritonitis, pneumonia (52)	600 mg bid Short duration IV (10 min to 2 h infusion) or oral Multiple doses	57.66 ^b^	77.03 ^b^	92.64 ^b^	V_c_, V_p_, CL, Q
Vancomycin							
Yamamoto et al. (2009) [63]	POP PK (NONMEM)	Healthy subjects (6)	500–1000 mg Over 1 h infusion Multiple doses	21.7 ± 2.0 (20–25)	60.3 ± 3.7 (55.2–64.2)	89.3 ± 10.4 (76.7–106.5)	V_c_, V_p_, CL, Q
Healy et al. (1987) [64]	Non-POP PK (NA)	Healthy subjects (11)	500 mg Q6 h or 1 g q12 h 1 h infusion Multiple doses	24.7 ± 2.1 (NI)	66.5 ± 11.2 (NI)	110 ± 19.3 (NI)	V_c_, V_ss_, CL
Shen et al. (2018) [65]	POP PK (NONMEM)	Adult patients (380)	No more than 2 g/day Duration of infusion: NI Multiple doses	61.35 ^b^	61.75 ^b^	86.91 ^b^	V_d_, CL
Sanchez et al. (2010) [66]	POP PK (NONMEM)	Hospitalised patients ^a^ (141)	1628 g/day Q6–48 h Duration of infusion: NI Multiple doses	55 ± 14.58 (NI)	73.2 ± 17.48 (NI)	76.13 ^e^	V_c_, V_p_, CL, Q
Dedkaew et al. (2015) [67]	POP PK (NLME)	ICU patients with bacteraemia, pneumonia, SSTI, meningitis, others (138)	1 g Q12 h or Q24 h 1 to 2 h infusion Multiple doses	65.7 ± 17.6 (18–97)	62.1 ± 13.7 (31.7–105)	54.5 ± 29.1 (10.03–105)	V_c_, V_p_, CL, C_min_
Roberts et al. (2011) [68]	POP PK (NONMEM)	ICU patients with sepsis (206)	LD = 750–1000 mg 0.5 h infusion MD = 2000–3000 mg 24 h infusion	58.1 ± 14.8 (NI)	74.8 ± 15.8 (NI)	90.7 ± 60.4	V_d_, CL

* Extracted PK parameters from the studies (more PK parameters are presented in the studies); ^a^ no information about detailed diagnosis; ^b^ converted from the median value; ^c^ average value from more than one subgroup; ^d^ presented as 4 h CL_Cr_; ^e^ calculated from Se_Cr_; ^#^ group 1: patients with preserved renal function (*n* = 8); group 2: patients with renal dysfunction (*n* = 9). POP PK = population pharmacokinetics; Non-POP PK = non-population pharmacokinetics; CAP = community-acquired pneumoniae; VAP = ventilator-acquired pneumonia; IAI = intra-abdominal infection; UTI = urinary tract infection; SSTI = skin and soft tissue infection; ARDS = acute respiratory distress syndrome; OD = once daily; bid = bis in die (two times a day); tid = ter in die (three times a day); Q6 h = every six hours; Q8 h = every eight hours; Q12 h = every 12 h; Q24 h = every 24 h; Q48 h = every 48 h; q8–12 h = every eight to 12 h. NI = no information; NA = not applicable; LD = loading dose; MD = maintenance dose; CoI = continuous infusion; IV = intravenous; V_c_ = volume distribution of central compartment; V_p_ = volume distribution of peripheral compartment; V_p2_ = volume distribution at second peripheral compartment; V_p3_ = volume distribution at third peripheral compartment; V_d_ = volume distribution total; V_dss_ = volume distribution at steady state; CL = clearance total; CL_ren_ = renal clearance; CL_non-ren_ = non-renal clearance; CL_ss_ = clearance at steady state; Q = intercompartmental clearance; K_CP_ = intercompartmental transfer rate from central to peripheral compartment; K_PC_ = intercompartmental transfer rate from peripheral to central compartment; K_a_ = absorption rate constant; t_1/2_ = half-life; C_max_ = maximum concentration; T_max_ = time to reach maximum concentration; AUC_0–24_ = area under the curve for the 24 h interval; AUC_0–12_ = area under the curve for the 12 h interval; K_e_ = elimination rate constant.

**Table 2:** Additional significant covariates of pharmacokinetic parameters of antimicrobials.

Antibiotics	PK Parameters					
	V_d_			CL		
	Patient Characteristics	Clinical Condition	Clinical Parameters	Patient Characteristics	Clinical Condition	Clinical Parameters
Meropenem	-	-	-	Age [42]	-	-
Imipenem	-	-	Serum albumin [50]	-	-	-
Doripenem	-	-	-	Weight [51]	-	-
Linezolid	Body surface area [60]	Peritonitis (469)	-	Age [60]; body surface area [60]	Severe liver cirrhosis [59]; acute respiratory distress syndrome [62]	Fibrinogen and lactate [62]
Vancomycin	Age [65,66]; Body weight [68]	Patients’ status (healthy or patients) [63]; type of infectious diseases [63]	-	-	-	-

**Table 3:** Pharmacokinetic differences between Asian and Non-Asian population groups.

Antimicrobials	Population	Comp (*n*)	V_c_ (L/kg)	V_p_ (L/kg)	CL (L/h/kg)	t_1/2_ (h)	Q (L/h)	K_CP_ (/h)	K_PC_ (/h)
Meropenem	Asian healthy subjects [39]	One (14)	Dose 1 g = 0.20 Dose 0.5 g = 0.16	NI	Dose 1 g = 0.22 Dose 0.5 g = 0.21	Dose 1 g = 0.64 Dose 0.5 g = 0.54	NI	NI	NI
	Comparator healthy subjects [40]	Two (6–18)	Dose 1 g = 0.19 ^Ϯ^ Dose 0.5 g = 0.18 ^Ϯ^	Dose 1 g = 0.06 ** Dose 0.5 g = 0.06 **	Dose 1 g = 0.25 ^Ϯ^ Dose 0.5 g = 0.24 ^Ϯ^	NI	NI	1.21	4.03
	Asian non-ICU patients [41]	One (1)	0.64 ^Ϯ^	NI	0.21 (IIV = 52.1%)	NI	NI	NI	NI
	Comparator non-ICU patients [42]	Two (1–12)	0.15 (IIV = 14.3%) V_d-tot_ = 0.32	0.17 (IIV = 10.2%)	0.2 (IIV = 11.8%)	NI	18.6 (IIV = 29.3%)	NI	NI
	Asian ICU patients [43]	Two (10)	0.16 ^a,Ϯ^	0.19 **	0.08 ^a,b,Ϯ^	NI	NI	1.85 ^Ϯ^	1.53 ^Ϯ^
	Comparator ICU patients [44]	One (1–2)	0.34 (IIV) = 71.2%)	NI	0.04 (IIV = 59.3%)	NI	NI	NI	NI
Imipenem	Asian healthy subjects [45]	Non-comp (10)	0.5 h infusion = 0.16 2 h infusion with dose 0.5 g = 0.16 2 h infusion with dose 1 g = 0.19	NI	0.5 h infusion = 0.14 2 h infusion with dose 0.5 g = 0.15 2 h infusion with dose 1 g = 0.14	0.5 h infusion = 1.32 2 h infusion with dose 0.5 g = 1.02 2 h infusion with dose 1 g = 2.42	NI	NI	NI
	Comparator healthy subjects [46]	Two (8–12)	Dose 0.5 g = 0.14 V_d-tot_ = 0.15 Dose 1 g = 0.13 V_d-tot_ = 0.14	Dose 0.5 g = 0.05 ** Dose 1 g = 0.06 **	Dose 0.5 g = 0.16 Dose 1 g = 0.15	Both dosing regimens = 1.0	NI	Dose 0.5 g = 0.8 Dose 1 g = 1.0	Dose 0.5 g = 2.2 Dose 1 g = 2.3
	Asian non-ICU patients [47]	Two (6–7)	0.19 (IIV = 18.9%) V_d-tot_ = 0.25	0.06 ^Ϯ^	0.14/0.07/0.06 ^c^ (IIV for CL renal = 34.1%; for CL non-renal = 29.4%)	NI	3.18 ^Ϯ^	NI	NI
	Comparator non-ICU patients [48]	Non-comp (6)	0.33	NI	0.17	1.6	NI	NI	NI
	Asian ICU patients [49]	Non-comp (4)	0.51/0.54 ^d^	NI	0.36/0.39 ^d^	0.98/0.97 ^d^	NI	NI	NI
	Comparator ICU patients [50]	Two (3–6)	0.26 (IIV = 48%) V_d-tot_ = 0.38	0.12 ^Ϯ^	0.16 (IIV = 48%)	NI	12.2 ^Ϯ^	NI	NI
Doripenem	Asian healthy subjects [51]	Two (12)	0.26 V_d-tot_ = 0.31 (IIV = 35.3%)	0.05	0.36 (IIV = 31.6%)	1.01 ^Ϯ^	1.83 ^Ϯ^	NI	NI
	Comparator healthy subjects [52]	Two (1–2)	0.13 (IIV = 14.4%) V_d-tot_ = 0.21	0.08 (IIV = 10.4%)	0.21 (IIV = 13.2%)	0.95 ^Ϯ^	9.69 ^Ϯ^	NI	NI
	Asian non-ICU patients [53]	One (4)	0.28 (IIV = 47.3%)	NA	0.11 (IIV = 55%)	NI	NI	NI	NI
	Comparator non-ICU patients [54]	Two (3–4)	0.19 ^Ϯ^	0.16 **	0.16 ^Ϯ^	NI	NI	4.7	5.7
	Asian ICU patients [55]	Two (6–8)	0.22 (IIV = 62%) V_d-tot_ = 0.47	0.25 (IIV = 73.3%)	0.14 (IIV = 56.7%)	NI	36.3 ^Ϯ^	NI	NI
	Comparator ICU patients [56]	Two (4–5)	0.29 (IIV = 93.7%) V_d-tot_ = 0.55	0.25 (IIV = 62.6%)	0.25 (IIV = 52.8%)	NI	23.3 ^Ϯ^	NI	NI
Linezolid	Asian healthy subjects [57]	Non-comp Single dose (13)	IV = 0.67 Oral = 0.65	NA	IV = 0.10 Oral = 0.11	IV = 4.37 Oral = 4.33	NI	NI	NI
	Comparator healthy subjects [58]	Non-comp Single dose ^e^ (36 over 11–16 days)	IV 625 mg = 0.58 Oral 625 mg = 0.61	NI	IV 625 mg ^c^ = 0.10/0.03/0.07 Oral 625 mg ^c^ = 0.09/0.03/0.06	IV 625 mg = 4.40 Oral 625 mg = 4.87	NI	NI	NI
	Asian non-ICU patients [59]	One (1–5)	0.59 (IIV = 35.8%)	NA	0.05 (IIV = 30.6%)	NI	NI	NI	NI
	Comparator non-ICU patients [60]	One (2–3)	0.58 (IIV = 17.8%)	NA	0.07 (IIV = 49.9%)	NI	NI	NI	NI
	Asian ICU patients [61]	Two (6)	Group 1 ^#^ = 0.34 Group 2 ^#^ = 0.37 (IIV = 32.3%) V_d-tot_ for: Group 1 ^#^ = 0.73 Group 2 ^#^ = 0.79	Group 1 ^#^ = 0.39 Group 2 ^#^ = 0.42 (IIV = 12.3%)	Group 1 ^#^ = 0.11 Group 2 ^#^ = 0.04 (IIV = 44.8%)	NI	26.4	NI	NI
	Comparator ICU patients [62]	Two (32 over 4 days)	0.19 ^f^ (IIV = 37%) V_d-tot_ = 0.54	0.35 ^f,Ϯ^	0.10 ^f^ (IIV = 58%)	NI	67.7 ^f^	NI	NI
Vancomycin	Asian healthy subjects [63]	Two (7–8)	0.21 (IIV = 18.2%)	0.65 (IIV = 72.8%)	0.06 (IIV = 37.5%)	NI	8.81 (IIV = 19.2%)	NI	NI
	Comparator healthy subjects [64]	Three (11–17)	Dose 0.5 g = 0.14 Dose 1 g = 0.11	NI	Dose 0.5 g = 0.08 Dose 1 g = 0.08	Dose 0.5 g = 8.1 Dose 1 g = 7.7	NI	NI	NI
	Asian non-ICU patients [65]	One (2)	0.73 (IIV = 24.8%)	NI	0.06 (IIV = 12.5%)	NI	NI	NI	NI
	Comparator non-ICU patients [66]	Two (1–2)	0.28 ^Ϯ^ V_d-tot_ = 0.72	0.44 (IIV = 6.8%)	0.03/0.04 ^g^ (IIV = 24.5%)	NI	8.13	NI	NI
	Asian ICU patients [67]	Two (2–8)	0.40 (IIV = 46%) V_d-tot_ = 0.79	0.39 ^Ϯ^ (IIV = 36.5%)	0.05 (IIV = 14%)	NI	NI	NI	NI
	Comparator ICU patients [68]	One (2–3)	1.53 (IIV = 37.4%)	NA	0.06 (IIV = 38.9%)	NI	NI	NI	NI

^a^ Calculated with assumption that the mean of body weight was 60 kg; ^b^ calculated from Ke; ^c^ Presented the value of CL_Tot_/CL_renal_/CL_non-renal_; ^d^ value for patients with renal infections/non-renal infections; ^e^ PK data after multiple-dose administration were not presented; ^f^ converted from the median value; ^g^ value for female/male subjects. ^#^ Group 1: patients with preserved renal function (*n* = 8); group 2: patients with renal dysfunction (*n* = 9); ^Ϯ^ the variance between subjects was not presented; ** V_p_ was predicted by considering the value of V_c_, K_CP_, K_PC_. *n* = Number of blood samples; NI = no information; IV = intravenous; Non-comp = non-compartmental analysis; V_c_ = volume distribution of central compartment; V_p_ = volume distribution of peripheral compartment; V_d-tot_ = volume distribution total; V_dss_ = volume distribution at steady state; CL = clearance total; CL_ren_ = renal clearance; CL_non-ren_ = non-renal clearance; CL_ss_ = clearance at steady state; Q = intercompartmental clearance; K_CP_ = intercompartmental transfer rate from central to peripheral compartment; K_PC_ = intercompartmental transfer rate from peripheral to central compartment; t_1/2_ = half-life; K_e_ = elimination rate constant.

**Table 4:** The relevance of inter-ethnic differences to the pharmacokinetic parameters of each antimicrobial.

Antimicrobials	PK Parameters	
	V_d_	CL
Meropenem [39,40,41,42,43,44]	Were not observed	Were not observed
Imipenem [45,46,47,48,49,50]	Were not observed	Were not observed
Doripenem [51,52,53,54,55,56]	Were observed Indicating: Asians > non-Asians [62,63]	Were not observed
Linezolid [57,58,59,60,61,62]	Were not observed	Were not observed
Vancomycin [63,64,65,66,67,68]	Were not observed	Were not observed

## Supplementary Materials

The following supporting information can be downloaded at https://www.mdpi.com/article/10.3390/antibiotics12050803/s1. Table S1: PRISMA items checklist for systematic review of inter-ethnic pharmacokinetic differences between Asian and non-Asian adult populations. Table S2: PRISMA checklist for abstract for systematic review of inter-ethnic pharmacokinetic differences between Asian and non-Asian adult populations. Table S3: Search terms in systematic review of inter-ethnic pharmacokinetic differences between Asian and non-Asian adult populations. Table S4: Reporting quality of pharmacokinetic studies included in the final analysis.

## Abbreviations

CL	clearance
CL_Cr_	creatinine clearance
CL_non-ren_	non-renal clearance
CL_ren_	clearance renal
C_max_	maximum concentration in a dosing interval
C_max,ss_	maximum concentration at pharmacokinetic steady state
C_min_	minimum concentration in a dosing interval
CYP450	cytochrome P450
ECMO	extracorporeal membrane oxygenation
ICU	intensive care unit
IIV	interindividual variability
IV	intravenous
K_CP_	the rate constant from the central compartment to the peripheral compartment
K_e_	elimination rate constant
K_PC_	the rate constant from the peripheral compartment to the central compartment
L	litre
L/h	litre per hour
L/kg	litre per kilogram body weight
L/h/kg	litre per hour per kilogram body weight
MIC	minimum inhibitory concentration
PK/PD	pharmacokinetic/pharmacodynamic
PK	pharmacokinetic
Q	intercompartmental clearance
RRT	renal replacement therapy
Se_Cr_	serum creatinine
t_1/2_	half-life
TDM	therapeutic drug monitoring
V_c_	volume of distribution in central compartment
V_d_	volume of distribution
V_dss_	volume of distribution at steady state
V_d-tot_	volume of distribution total
V_p_	volume of distribution in peripheral compartment
